# Identification of Differential Drive Robot Dynamic Model Parameters

**DOI:** 10.3390/ma16020683

**Published:** 2023-01-10

**Authors:** Michał Siwek, Jarosław Panasiuk, Leszek Baranowski, Wojciech Kaczmarek, Piotr Prusaczyk, Szymon Borys

**Affiliations:** Faculty of Mechatronics, Armament and Aerospace, Military University of Technology, Kaliskiego 2 Street, 00-908 Warsaw, Poland

**Keywords:** mobile robot, ROS, AGV, identification, adaptation, trajectory tracking

## Abstract

The paper presents the identification process of the mathematical model parameters of a differential-drive two-wheeled mobile robot. The values of the unknown parameters of the dynamics model were determined by carrying out their identification offline with the Levenberg-Marguardt method and identification online with the Recursive least-squares method. The authors compared the parameters identified by offline and online methods and proposed to support the recursive least squares method with the results obtained by offline identification. The correctness of the identification process of the robot dynamics model parameters, and the operation of the control system was verified by comparing the desired trajectories and those obtained through simulation studies and laboratory tests. Then an analysis of errors defined as the difference between the values of reference position, orientation and velocity, and those obtained from simulations and laboratory tests was carried out. On itd basis, the quality of regulation in the proposed algorithm was determined.

## 1. Introduction

**Figure 1:**
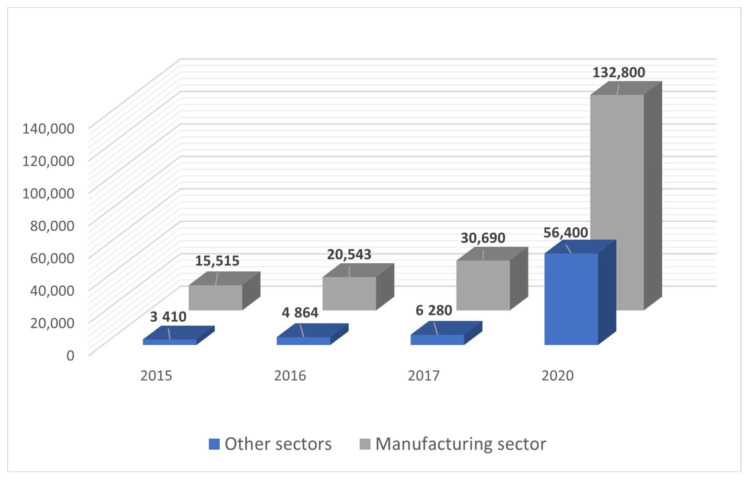
The number of mobile robots used in industry [2].

By Industry 4.0 standards, internal transportation is becoming one of the most important areas undergoing transformation. Due to the increasingly scarce labor resources and the increasing cost of transporting products in the production process, transportation is considered in the category of losses. Therefore, there is a need to minimize the costs associated with the internal transportation by reducing the distances and times of product movement. One of the solutions to this problem is the introduction of autonomous transport robots that will accurately, quickly, and safely carry out the tasks assigned to them. Automated guided vehicles are mainly used to support material handling operations—in the warehouse, in production and at the intersection of these areas [1]. This can involve a range of tasks, from the timely delivery of parts to the production line to round-the-clock transportation. Full automation of the production process makes it possible to ensure smooth manufacturing of products along the entire process line. Therefore, in line with the idea of Industry 4.0, more and more often, mobile cart units or AGV (Automated Guided Vehicle) robots appear at the end of the production line to transport and manage products. The market for mobile robots is still at an early stage of development.However, for several years there has been an exponential increase in the implementation of transport mobile robots in the industrial sector.This is due to the fact that more and more companies are noticing the possibilities of this type of vehicles to automate internal transport (Figure 1).

When deciding to integrate mobile robots into a production process at a company, it is necessary to define the efficiency of the transport system based on the following assumptions: the number of robots, the number of logistics points, the location of the navigation paths along which they will move and the frequency of changeovers.

The first automatic carts implementing operator-free driving appeared in the 1950s. These were single implementations primarily in the automotive industry. Over time, the advantages of AGV deployments were increasingly recognized and automatic vehicles began to appear in other industries as well, especially in applications related to in-plant logistics. Since then, AGV robot technology has been developing rapidly, as indicated by the fact that there are several types of AGVs [3,4]. The main characteristic of AGV robots is their driving system. One can distinguish:

- three-wheeled systems (two independent driven wheels, one non-driven castor wheel);
- four-wheeled systems:
  – two independent driven standard wheels mounted on the front of the robot and two castor wheels mounted on the rear of the robot;
  – four independently controlled Swedish wheels;
- six-wheeled systems (two independent driven standard wheels mounted in the center of the robot frame and four castor wheels mounted on the corners of the robot frame).

All vehicles for automatic load handling are regulated by ISO 3691-4:2020 and called automated guided vehicle (AGV) [5]. This standard distinguishes specific names for different types of AGV introduced by manufacturers of these machines. At the beginning, the main division into AGV and AMR (Autonomous Mobile Robots) can be distinguished. AGV technology refers to automated vehicles that move indoors along physically defined paths, requiring the installation of lines or navigation points. This imposes the need to navigate only along a designated path. In case of encountering an obstacle, it requires stopping and waiting for its removal. Changing the path requires changing the navigation line and other markings. An AMR solution involves autonomous vehicles, often called intelligent vehicles, equipped with 2D or 3D scanners, moving based on a self-created map along a self-determined path between destination points, with the ability to avoid obstacles. The use of AMR technology allows for improved production flexibility.The robot itself determines the optimal route for a given task [6]. Changing the tasks or destinations assigned to it is very quick and does not require physical interference in the space in which the robot moves.

This work proposes a control system for the tracking motion of a mobile robot with two differentially controlled drive wheels and two support wheels. The control system was realized using a kinematic and dynamic controller, where the input and output signals are linear and angular velocity. In the presented approach, the problem is the parameters of the mathematical model of the robot dynamics, which are not available to the user. Therefore, their identification is carried out. In this work, the authors performed offline identification using the Levenberg-Marguardt method based on a pre-recorded trajectory, and online identification using the Recursive least-squares (RLS) method while moving along a desired trajectory. To verify the correctness of the identified parameters, simulation studies were carried out in the MATLAB/Simulink environment, followed by laboratory tests using a real robot. During the tests, the effect of random initial parameters in the identification process on the accuracy of trajectory tracking was investigated. The parameters identified by online and offline methods were compared. The novelty of proposed method is the usage of the parameters identified by the Levenberg-Marguardt method as the initial parameters in the online identification. The effect of combination of online and offline methods on the accuracy of tracking the desired trajectory was investigated.

In Section 2, the authors presented related works. Section 3 presents the selected research object for which the control system, kinematic and dynamic model were developed. The Section 4 contains the results of the identification process, the design of the control system, and the results of trajectory tracking considering the identified parameters. In Section 5, the authors summarized the work carried out.

## 2. Related Works

AGV robots are a good solution for plants that are characterized by low variability of the production process and not very large distances between logistic destinations. In the case of frequent changes in the production process, dynamically changing environment in the plant (e.g., moving workers) [7], disturbances [8,9] and obstacles [10], a safe and flexible control system is required to allow for environmental changes [11,12,13] or reprogramming of the carts for other tasks. Searching for the most appropriate path planning algorithm according to user requirements can be challenging given the large number of examples existing in the literature [14,15,16,17,18,19]. Furthermore, in the case of AGV robots, the precision of the final positioning is crucial for successful loading and unloading of transported goods in typical industrial applications [20]. Designers of robotics systems use advanced tools to assist in the design process of control systems [21,22,23,24,25] to provide the expected functionality of the robot. The working environment has a particular impact on the change in the operating parameters of the robot. Especially when it is difficult [26,27] such as dusty rooms or industrial and sewage pipelines or mines [28].

A way to obtain unknown values of robot model parameters is to identify them. The work [29,30,31,32] presents a methodology for offline identification based on an inverse dynamics model of robots. The offline identification of the dynamics parameters is sufficient only in the case of robots that will not be expanded with additional modules or will not transport loads. In the case of AGV robots transporting loads, their mass and moment of inertia change, which means that the parameters of the model are not constant. When designing the control algorithm, it is necessary to take into account the possibility of changing the model parameters based on their identification using adaptive methods (online identification) [33,34,35,36,37,38]. The most widely used adaptive methods is the Recursive least squares (RLS) method, as presented in papers [39,40]. The example is for trajectory tracking of an autonomous ground vehicle. To estimate the unknown parameters of the system, the authors of [39] proposed a set-membership-based estimator that provides a nonincreasing estimation error. In the proposed predictive controller (MPC) scheme, the authors used the RLS method to improve prediction accuracy by introducing a robustness constraint on the algorithm to parametric and additive uncertainties. The robustness factor is determined offline based on a set of invariant parameters. The size of this set is updated online. The proposed method is a bit computationally complex, so it requires a high-performance on-board computer. Another example of the use of the RLS method is the work [41], whose authors presented a positioning system for a mobile robot using a stereoscopic camera, an IMU, and an Ultra Wideband (UWB) network containing five positioning markers. The system can be extended with additional positioning markers, whose unknown position in the robot’s environment is estimated using the RLS method and Kalman filter. The proposed system works well in general locating the robot in an unknown environment. However it comes with a large positioning error, which unfortunately is not acceptable for transportation tasks. An interesting approach, consistent with the authors of this work, was demonstrated by the authors of papers [41,42,43,44]. They presented the problem of trajectory tracking by a nonholonomic mobile robot without a global positioning system. The main difficulty pointed out in this work is localizing the robot using only its on-board sensors. The authors proposed an adaptive trajectory tracking controller without orientation and velocity measurements. Their method is not based on the estimation of the robot’s position and velocity from the image of an omnidirectional camera mounted on the robot. The paper [43] presents a fusion of an inertial sensor of six degrees of freedom (6-DoF) which comprises the 3-axis of an accelerometer and the 3-axis of a gyroscope, and a vision to determine a low-cost and accurate position for an autonomous mobile robot. For vision, a monocular vision-based object detection algorithm speeded-up robust feature (SURF) and random sample consensus (RANSAC) algorithms were integrated and used to recognize a sample object in several images taken. In contrast to the work of [42], the authors of this work, present a control concept that not use robot-mounted peripheral devices such as laser scanners and cameras. The tracking control concept is based only on odometry data and inertial measurements.

## 3. Materials and Methods

**Figure 2:**
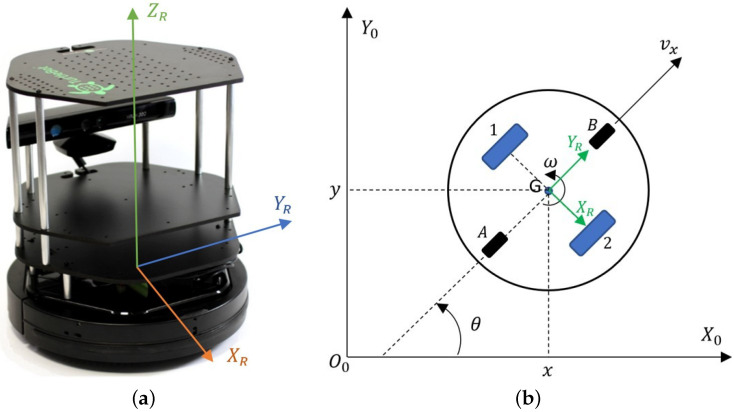
(**a**) Isometric view of the TURTLEBOT 2 robot with the coordinate system; (**b**) kinematic structure to determine the kinematic model.

The considerations presented in this paper include the TURTLEBOT 2 laboratory robot (Figure 2) [45], which is a low-cost training set, used to building and testing wheeled mobile robot control systems. The platform is equipped with a two-wheeled differential drive and basic sensors such as a laser scanner, a 640 × 480 resolution RGB video camera (used for image processing, color, and texture recognition), an IR camera (depth information processing, distance measurement in the range of 0.4–6.5 m), four directional microphones, and an accelerometer. An advantage of using the robot in this task is its open control system based on ROS (Robot Operating System) [46,47,48], which allows quick launch of the platform and integration with sensors [45]. In addition, the drive system of the TURTLEBOT 2 robot is the basis for expansion to the drive systems discussed in the introduction, commonly used in industrial AGVs. Transferring and adapting the control system to another chassis will be extremely fast and simple.

### 3.1. Kinematic and Dynamic Robot Model

**Figure 3:**
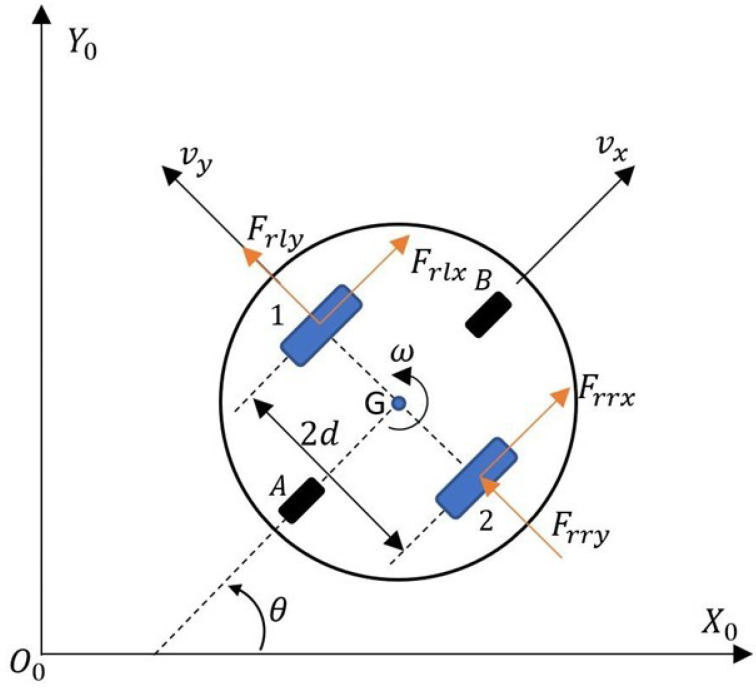
TURTLEBOT 2 robot schematic for developing the dynamics model.

In order to determine the kinematic and dynamic model of the TURTLEBOT 2 robot, the autors used the diagrams shown in Figure 2 and Figure 3.

The $0_{0}X_{0}Y_{0}$ coordinate system shown in Figure 2b is a global, fixed reference system. The *x*, *y* coordinates define the positions and the angle θ define the orientation of the robot in the global coordinate system. A movable coordinate system $GX_{R}Y_{R}$ with an origin at the geometric center of the robot *G*, is associated with the base of the robot. The vector ν determines the linear velocity, while ω determines the angular velocity of the robot. The numbers 1 and 2 denote the drive wheels, while the letters *A* and *B* denote the supporting wheels. The kinematics of the mobile robot TURTLEBOT 2 is described based on [49,50,51,52] by Equation (1) in the form in which the control signals are the linear and angular velocities of the robot:

(1) $$\left[\begin{array}{c} \dot{x}\\ \dot{y}\\ \dot{\theta } \end{array}\right]=\left[\begin{array}{cc} cos\theta & 0\\ sin\theta & 0\\ 0 & 1 \end{array}\right]\left[\begin{array}{c} v\\ \omega \end{array}\right]$$

While analyzing the literature in view of control systems, it can be noted that most of the proposed controllers performing the trajectory tracking task consider only the kinematics of the robot. This is an acceptable solution, but in the case of tasks requiring high positioning accuracy and moving at high speed, it is necessary to consider the dynamics of the robot in the control system. In the literature, dynamic models of robots are popular, taking into account torque or motor voltages as steering signals [53,54,55,56]. A much better solution is to use a dynamics model that takes the linear and angular velocities of the robot as control signals [57,58]. This is how commercially available mobile robots are usually controlled. Figure 3 shows a schematic of the TURTLEBOT 2 robot used to determine the dynamics model.

The point *G* (Figure 3) is not only the robot’s center of mass and center of rotation, but also the tracking point for the desired trajectory. The robot’s velocities are denoted as: $\nu_{x}$—forward velocity, $\nu_{y}$—lateral velocity, ω— angular velocity. In Figure 3, the driving forces of the robot are denoted as $F_{rrx}$, $F_{rry}$—the longitudinal and lateral force of the right wheel and $F_{rlx}$, $F_{rly}$—the longitudinal and lateral force of the left wheel. The dynamics model of the robot was formulated by performing a law of conservation of linear and angular momentum, in the coordinate system associated with the robot, following [59]:

(2) $$\begin{array}{c} \sum F_{x}=F_{rlx}+F_{rrx}=m\left({\dot{v}}_{x}-v_{y}\omega \right)\\ \sum F_{y}=F_{rly}+F_{rry}=m\left({\dot{v}}_{y}-v_{x}\omega \right)\\ \sum M_{z}=I_{z}\omega =\frac{d}{2}\left(F_{rrx}-F_{rlx}\right) \end{array}$$

where:

*m*—mass of the robot,

$I_{z}$—moment of inertia of the robot with respect to the axis passing through the point *G*.

The transformation of the system of Equation (2) (see Appendix A) is the dynamic model of the TURTLEBOT 2 robot as presented:

(3) $$\left[\begin{array}{c} {\dot{v}}_{x}\\ \dot{\omega } \end{array}\right]=\left[\begin{array}{c} -\frac{\sigma_{3}}{\sigma_{1}}v_{x}\\ -\frac{\sigma_{4}}{\sigma_{2}}\omega \end{array}\right]+\left[\begin{array}{cc} \frac{1}{\sigma_{1}} & 0\\ 0 & \frac{1}{\sigma_{2}} \end{array}\right]\left[\begin{array}{c} v_{xd}\\ \omega_{d} \end{array}\right]$$

The model coefficients $\sigma_{1}$…$\sigma_{4}$ contain parameters that are very difficult for a laboratory robot user to obtain. These coefficients are functions of the physical parameters of the robot related to drives, gears, internal controls, friction, and are usually not even included in the manuals or technical sheets of the equipment, so their identification was carried out.

### 3.2. Offline Identification of Robot Dynamic Model Parameters

Before identifying model parameters, it should be checked whether any of the model parameters can be written as a linear combination of two others. In such a case, it would be possible to write the robot dynamics model with the lower number of parameters. Analyzing the developed mathematical model of the robot, the linear independence between the parameters $\sigma_{1}$…$\sigma_{4}$ is not clearly visible, because some physical parameters affect more than one parameter $\sigma_{n}$. A detailed analysis is necessary, which was carried out in the work [58] showing that the model parameters $\sigma_{1}$ to $\sigma_{4}$ are independent, that is, they cannot be written as their linear combination. Ultimately, it is necessary to carry out identification of each of the parameters $\sigma_{1}$…$\sigma_{4}$.

Offline identification of the parameters of the robot’s dynamics model was carried out using the Levenberg-Marguardt method and measurements of the real test trajectory made by the robot at known constant values of linear and angular velocity. Identification is an iterative process, so in order to perform the first iteration, initial starting values must be entered at the beginning.

To evaluate the impact of starting parameter error on identification time and accuracy, the process was carried out for random initial values of the identified parameter. Starting parameters were described by a normal distribution with expectation value μ=1 variance $\sigma^{2}=1$ in the interval 〈0,1〉. The next step was to determine the mean value, the variance and the standard deviation from all samples of the identified parameter.

For the Levenberg-Marguardt method, the minimization criterion takes the form [60,61]:

(4) $${}_{d}^{min}{∥J\left(x\right)d+F\left(x\right)∥}_{2}^{2}$$

where J(x) is the Jacobian of the vector F(x), and *d* is the direction of the search for the minimum of the function determined according to the rule:

(5) $$\left[{J\left(x\right)}^{T}J\left(x\right)+\alpha I\right]d=-J\left(x\right)F\left(x\right),\;\;\;\;\;\;for\;\alpha \geq 0\;$$

In the identification process, 50 tests were carried out. Table 1 contains sample values of the identified parameters of the robot dynamics model.

The low values of the standard deviation of the identified parameters suggest the correctness of the identification process for the Levenberg-Marguardt method, which was verified in simulation and laboratory tests, as presented in Section 4.2, Section 4.3, Section 4.4 and Section 4.5.

### 3.3. Online Identification of Robot Model Parameters

In a dynamic system, there may be uncertainties in the robot parameters determined by offline methods; therefore, in [40,57,58], adaptation of the robot dynamics model parameters during motion was proposed. In the paper [58], two laws of adaptation of robot dynamics model parameters were presented. The system discussed in this paper uses parameter adaptation based on the expression:

(6) $$\dot{\hat{\sigma }}=\gamma^{-1}A^{T}\Delta -\gamma^{-1}\Gamma \hat{\sigma }$$

where $\gamma \in R^{4\times 4}$ is the gain matrix for parameter adaptation, and $\hat{\sigma }$ is the matrix of estimated parameters of the dynamics model, *A* is the matrix of velocities and accelerations (see Appendix B), and Δ is the matrix of lapses in the form:

(7) $$\Delta =\;\left[\begin{array}{c} v_{xd}\\ \omega_{d} \end{array}\right]-\left[\begin{array}{c} v_{x}\\ \omega \end{array}\right]$$

The adaptation law (6) by introducing a gain matrix $\Gamma \in R^{4\times 4}$ gives the possibility to disable the updating of the model parameters when the error is smaller than a preset limit.

In order to check the effect of unknown model parameters on the initial tracking error of the desired trajectory, two studies were conducted in which random values were used as initial parameters in the identification process. Then the values were replaced with values determined by the offline method.

**Figure 4:**
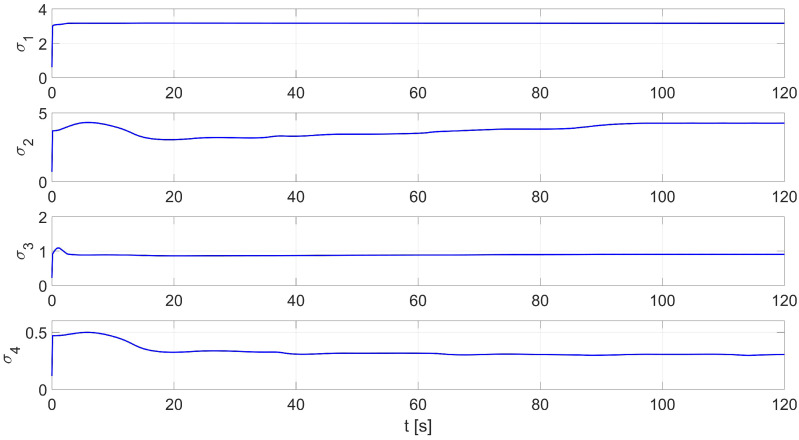
An example of the RLS identification process (test No. 1 from Table 2).

In the first study, online identification was carried out for random initial parameters described by a normal distribution with expectation value μ=1 and variance $\sigma^{2}=1$ in the interval 〈0,1〉. The simulation time was set at 120 s. An example of the distribution of the identified parameters $\sigma_{1}$…$\sigma_{4}$ is shown in Figure 4. The values of the starting parameters and those identified by the RLS method are shown in Table 2.

**Figure 5:**
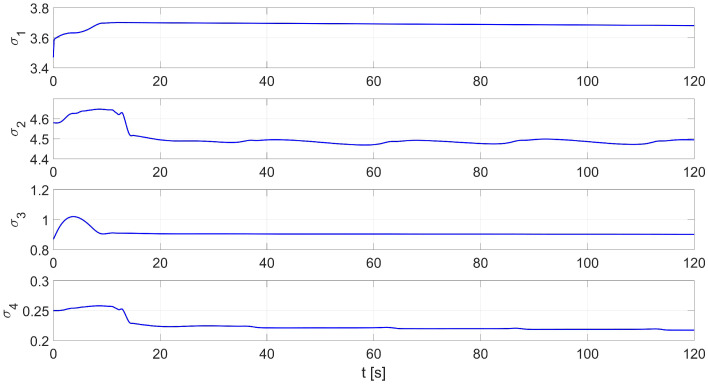
An example of the identification process using the Levenberg-Marguardt-assisted RLS method.

In the second test of the identification process by the RLS method, the parameters identified by the Levenberg-Marguardt method were used as initial parameters. Figure 5 shows the distribution of the identified parameters $\sigma_{1}$…$\sigma_{4}$. The values of the initial and identified parameters by the Levenberg-Marguardt-assisted RLS method are shown in Table 3.

## 4. Results

The correctness of the TURTLEBOT 2 robot’s tracking control algorithm and the correctness of the identification of the parameters of its mathematical dynamic model were checked first by simulation and then by implementation on a real robot. The tests were carried out for an 8-shape trajectory described by a system of equations:

(8) $$\left[\begin{array}{c} x_{d}\\ y_{d}\\ \theta_{d} \end{array}\right]=\left[\begin{array}{c} x_{0}+A_{x}\sin\left(\omega_{x}t\right)\\ y_{0}+A_{y}cos\left(\omega_{y}t\right)\\ atan2\left({\dot{y}}_{d},{\dot{x}}_{d}\right)+k\pi \end{array}\right]$$

where: $x_{0}=0$, $y_{0}=0$, $A_{x}=2$ [m], $A_{y}=2$ [m], $\omega_{x}=0.25\;\left[\frac{\mathrm{rad}}{s}\right]$, $\omega_{y}=0.125\;\left[\frac{\mathrm{rad}}{s}\right]$, k=0 if the robot moves forward k=1 if the robot moves backwards.

### 4.1. Kinematic and Dynamic Controller

The implementation of the trajectory tracking task by a robot is always associated with the appearance of errors—the difference between the reference position and the actual position [15,62]. For this purpose, a proportional controller with a feedback loop was proposed, developed based on [63]. Whose task is minimizing the position errors $e_{x},\;e_{y},\;e_{\theta }$ described by Equation (9). It is a transformation of the difference between the desired position and orientation, denoted as $x\left(t\right),\;y\left(t\right),\;\theta \left(t\right)$, and the real position and orientation, denoted as $x_{r}\left(t\right),\;y_{r}\left(t\right),\;\theta_{r}\left(t\right)$:

(9) $$\left[\begin{array}{c} e_{x}\left(t\right)\\ e_{y}\left(t\right)\\ e_{\theta }\left(t\right) \end{array}\right]=\left[\begin{array}{ccc} cos\theta (t) & sin\theta (t) & 0\\ -sin\theta (t) & cos\theta (t) & 0\\ 0 & 0 & 1 \end{array}\right]\left[\begin{array}{c} x\left(t\right)-x_{r}\left(t\right)\\ y\left(t\right)-y_{r}\left(t\right)\\ \theta \left(t\right)-\theta_{r}\left(t\right) \end{array}\right]$$

The kinematic controller proposed based on [63] takes the form:

(10) $$\left[\begin{array}{c} u_{kv}\left(t\right)\\ u_{k\omega }\left(t\right) \end{array}\right]=\left[\begin{array}{ccc} -k_{1} & 0 & 0\\ 0 & -sign\left(v_{r}\left(t\right)\right)k_{2} & -k_{3} \end{array}\right]\left[\begin{array}{c} e_{x}\left(t\right)\\ e_{y}\left(t\right)\\ e_{\theta }\left(t\right) \end{array}\right]$$

where $\nu_{r}\left(t\right)$—real linear velocity, $u_{k\nu }\left(t\right)$ and $u_{k\omega }\left(t\right)$ are the control signals of the kinematic controller (linear and angular velocity) and its gains $k_{1},k_{2},k_{3}$ are determined from the following equations [63]:

(11) $$\begin{array}{cc} \; & k_{1}=k_{3}=2\varepsilon \omega_{n}\left(t\right),\;\;\;\;\;\;for\;\varepsilon \in \left(0,1\right),\;b>0\\ \; & k_{2}=b*\left|v_{r}\left(t\right)\right|\\ \; & \omega_{n}\left(t\right)=\sqrt{{\omega_{r}}^{2}\left(t\right)+b{v_{r}}^{2}\left(t\right)} \end{array}$$

where:

ε—damping coefficient, $\omega_{n}\left(t\right)$—characteristic frequency, *b*—additional control coefficient, $\omega_{r}\left(t\right)$—real angular velocity.

The assumption for the development of the dynamic controller was to treat the control signals from the kinematics controller (linear and angular velocity) expressed by relation (10) as reference signals. The control law for the dynamic controller was developed based on the inverse dynamics (Appendix B), using the parametric model of robot dynamics (3) and the considerations presented in the paper [58]. The control law is described by the equation:

(12) $$\begin{array}{cc} \; & \left[\begin{array}{c} u_{dv}\left(t\right)\\ u_{d\omega }\left(t\right) \end{array}\right]=\left[\begin{array}{cc} \sigma_{1} & 0\\ 0 & \sigma_{2} \end{array}\right]\left[\begin{array}{c} \vartheta_{1}\\ \vartheta_{2} \end{array}\right]+\left[\begin{array}{cc} \begin{array}{cc} \begin{array}{c} 0\\ 0 \end{array} & \begin{array}{c} 0\\ 0 \end{array} \end{array} & \begin{array}{cc} \begin{array}{c} v_{r}\left(t\right)\\ 0 \end{array} & \begin{array}{c} 0\\ \omega_{r}\left(t\right) \end{array} \end{array} \end{array}\right]{\left[\begin{array}{cc} \begin{array}{ccc} \sigma_{1} & \sigma_{2} & \sigma_{3} \end{array} & \sigma_{4} \end{array}\right]}^{T}\\ \; & \vartheta_{1}=\;\dot{v}\left(t\right)+k_{v}\left(u_{kv}\left(t\right)-v_{r}\left(t\right)\right)\\ \; & \vartheta_{2}=\;\dot{\omega }\left(t\right)+k_{\omega }\left(u_{k\omega }\left(t\right)-\omega_{r}\left(t\right)\right) \end{array}$$

where $k_{\nu }$ and $k_{\omega }$ are gains, $u_{dv}\left(t\right)$ and $u_{d\omega }\left(t\right)$ are control signals of the dynamic controller.

### 4.2. Simulation Tests of the Control System Considering Parameters Identified Offline

**Figure 6:**
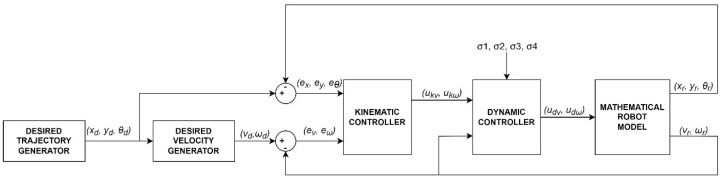
Block diagram of the tracking motion simulation of the TURTLEBOT 2 robot.

Simulation of the tracking motion of the TURTLEBOT 2 robot (Figure 6) was developed in MATLAB/Simulink environment, using the identified parameters of the robot model. The control system is composed of a:

- desired trajectory generator module that determines the position $x_{d}$, $y_{d}$ and orientation $\theta_{d}$,
- a generator of the desired linear velocity $v_{d}$ and angular velocity $\omega_{d}$,
- a kinematic controller described by Equation (10),
- a dynamic controller described by Equation (12),
- and a mathematical model of the robot described by Equations (1) and (3).

**Figure 7:**
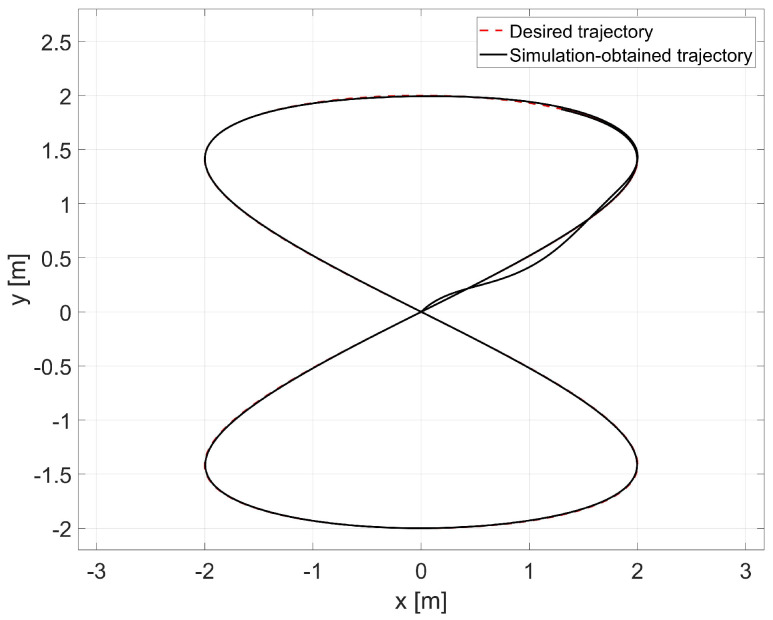
Comparison of the desired trajectory and that obtained by simulation for the Levenberg- Marguardt method.

**Figure 8:**
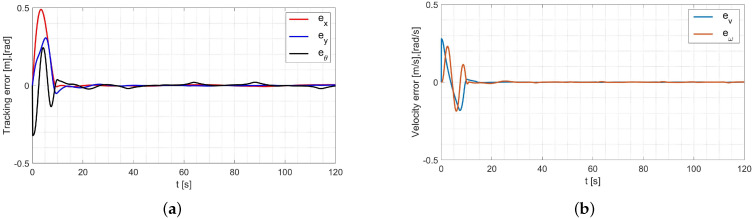
Errors for the Levenberg-Marguardt method: (**a**) reference trajectory tracking, (**b**) velocity.

In the simulation, the average values of the model parameters shown in Table 1 were used. A comparison of the desired trajectory and the simulation-obtained trajectory, using the parameters identified by the Levenberg-Marguardt method is shown in Figure 7. The position and orientation errors are shown in Figure 8a, while the velocity errors are shown in Figure 8b.

Based on the analysis of the simulation-determined errors for the Levenberg-Marguardt method (Figure 8), it can be concluded that the controller, developed based on the inverse dynamics with the parameters identified by the Levenberg-Marguardt method, realizes control in tracking motion with position, orientation and velocity errors no greater than:

- in the x-axis direction: $e_{x}=0.007\;\left[m\right]$,
- in the y-axis direction: $e_{y}=0.007\;\left[m\right]$,
- orientation: $e_{\theta }=0.021\;\left[\mathrm{rad}\right]$,
- linear velocity $e_{v}=0.001\;\left[\frac{m}{s}\right]$,
- angular velocity $e_{\omega }=0.007\;\left[\frac{\mathrm{rad}}{s}\right]$.

The regulation time for the case of an 8-shape trajectory tracking task is equal to $t_{r}\;\approx \;8.2\;\left[s\right]$.

### 4.3. Simulation Studies of the Control System with Online Identification

**Figure 9:**
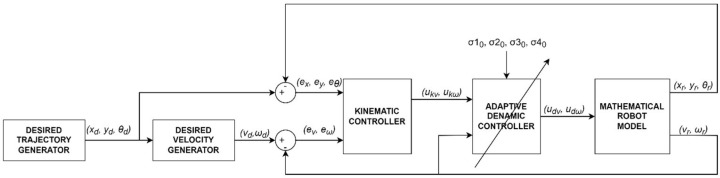
Block diagram of the TURTLEBOT 2 robot’s tracking motion simulation.

Online parameters identification of the robot dynamics model was carried out based on the tracking motion simulation scheme shown in Figure 9.

**Figure 10:**
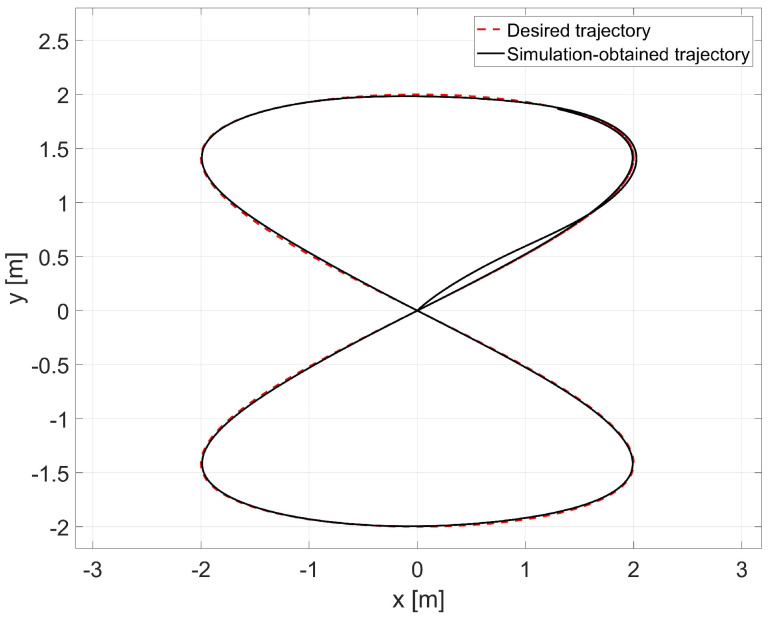
Comparison of reference and actual trajectories in a simulation test for the RLS method with random initial parameters.

**Figure 11:**
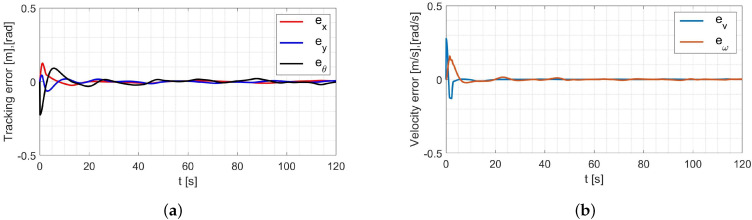
Errors for the RLS method with random initial parameters: (**a**) tracking the reference trajectory (**b**) velocity.

In this case, an adaptive dynamic controller using the RLS method described by Equation (6) was used. The simulation was first carried out for parameters with random values equal to $\sigma_{1}=0.52,\;\sigma_{2}=0.23,\;\sigma_{3}=0.49,\;\sigma_{4}=0.62$. A comparison of the desired and resulting trajectories is shown in Figure 10. The position and orientation errors are shown in Figure 11a, while the velocity errors are shown in Figure 11b.

Based on the analysis of the simulation-determined errors for the RLS method with random initial parameters (Figure 11), it can be concluded that the adaptive controller, realizes control in tracking motion with position, orientation and velocity errors no greater than:

- in the x-axis direction: $e_{x}=0.009\;\left[m\right]$,
- in the y-axis direction: $e_{y}=0.016\;\left[m\right]$,
- orientation: $e_{\theta }=0.022\;\left[\mathrm{rad}\right]$,
- linear velocity $e_{v}=0.001\;\left[\frac{m}{s}\right]$,
- angular velocity $e_{\omega }=0.015\;\left[\frac{\mathrm{rad}}{s}\right]$.

The regulation time for the case of an 8-shape trajectory tracking task is equal to $t_{r}\;\approx \;7.8\;[s$].

**Figure 12:**
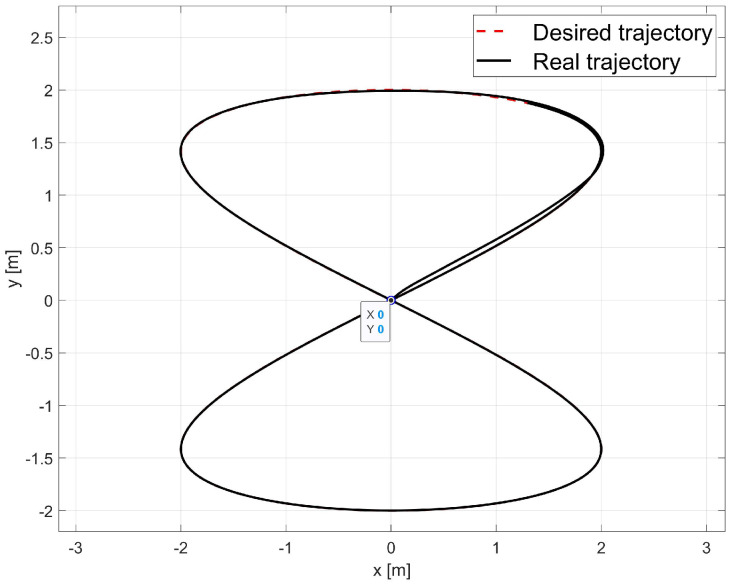
Comparison of the reference trajectory and the one obtained by simulation for the combination of RLS and Levenberg-Marguardt methods.

**Figure 13:**
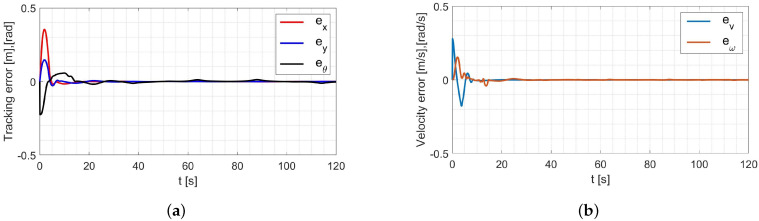
Errors for the RLS method with initial parameters determined by the Levenberg-Marguardt method: (**a**) tracking the reference trajectory (**b**) velocity.

Next, the random initial parameters were replaced with average values determined using the Levenberg-Marguardt method. A comparison of the desired trajectory and obtained trajectories from this simulation is shown in Figure 12. The position and orientation errors are shown in Figure 13a, while the velocity errors are shown in Figure 13b.

Based on the analysis of the simulation-determined errors for the RLS method with the initial parameters determined by the Levenberg-Marguardt method (Figure 13), it can be concluded that the adaptive controller, realizes control in tracking motion with position, orientation and velocity errors no greater than:

- in the x-axis direction: $e_{x}=0.005\;\left[m\right]$,
- in the y-axis direction: $e_{y}=0.002\;\left[m\right]$,
- orientation: $e_{\theta }=0.001\;\left[\mathrm{rad}\right]$,
- linear velocity $e_{v}=0.001\;\left[\frac{m}{s}\right]$,
- angular velocity $e_{\omega }=0.017\;\left[\frac{\mathrm{rad}}{s}\right]$.

The regulation time for the case of an 8-shape trajectory tracking task is equal to $t_{r}\;\approx \;4.8\;\left[s\right]$.

### 4.4. Laboratory Tests of the Control System with Parameters Identified Offline

**Figure 14:**
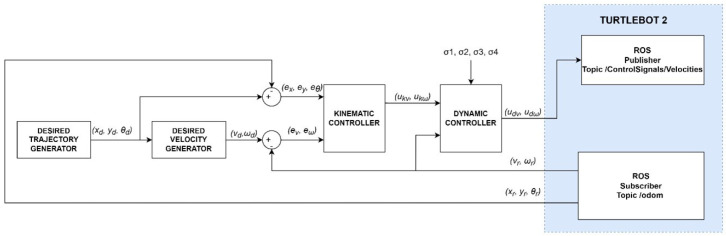
Block diagram of the real TURTLEBOT 2 robot’s tracking motion control system.

Verification of the identified parameters was carried out by laboratory tests of the tracking motion using a real robot TURTLEBOT 2. The test used a control system, including a kinematics (10) and dynamics (12) controller, implemented using the basic elements of the Simulink package. The Simulink/ROS library and two tools (Publisher—to publish a specific type of message in a declared communication node, and Subscriber—to receive messages from the robot system transmitted in a declared communication node) were used to communicate with the robot’s operating system. Using Publisher, control signals in the form of linear and angular velocity are sent to the robot system. Using Subscriber, position and orientation are received from the robot system. A block diagram of trajectory tracking by the real TURTLEBOT 2 robot, taking into account parameters identified by offline methods, is shown in Figure 14.

**Figure 15:**
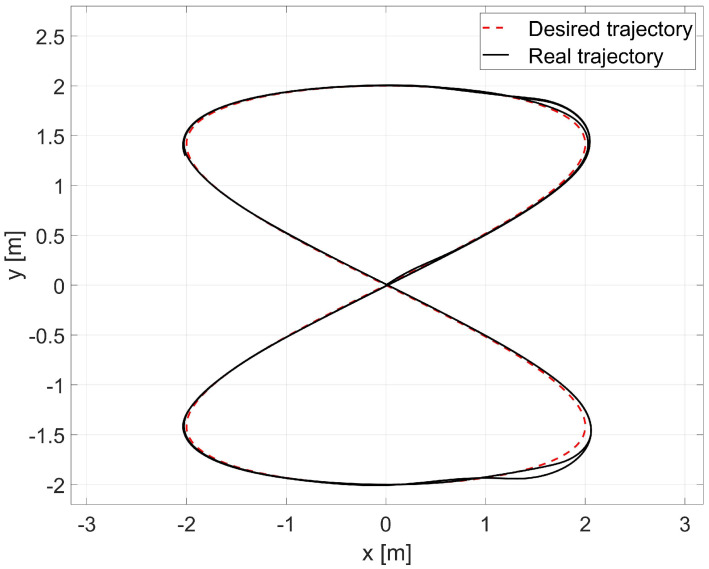
Comparison of reference and real trajectories in a laboratory test.

**Figure 16:**
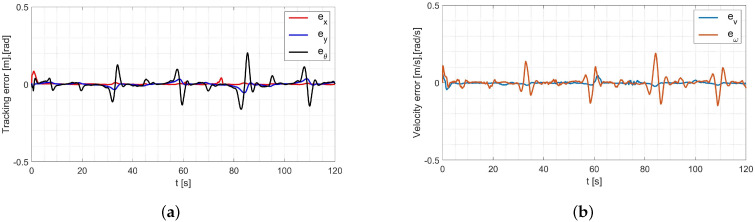
Errors for the Levenberg-Marguardt method: (**a**) tracking the reference trajectory (**b**) velocity.

The laboratory test used the average values of the model parameters shown in Table 1. A comparison of the reference and real trajectory for TURTLEBOT 2 robot with the parameters identified by the Levenberg-Marguardt method is shown in Figure 15, position and orientation errors are shown in Figure 16a, and velocity errors are shown in Figure 16b.

Based on the analysis of the errors determined in laboratory tests (Figure 16), it can be concluded that the controller, developed based on the inverse dynamics with the parameters identified by the Levenberg-Marguardt method, realizes control in tracking motion with position, orientation and velocity errors no greater than:

- in the x-axis direction: $e_{x}=0.033\;\left[m\right]$,
- in the y-axis direction: $e_{y}=0.046\;\left[m\right]$,
- orientation: $e_{\theta }=0.2\;\left[\mathrm{rad}\right]$,
- linear velocity $e_{v}=0.023\;\left[\frac{m}{s}\right]$,
- angular velocity $e_{\omega }=0.18\;\left[\frac{\mathrm{rad}}{s}\right]$.

The regulation time for the case of 8-shape trajectory tracking task is equal to $t_{r}\;\approx \;2.9\;\left[s\right]$.

### 4.5. Laboratory Tests of Control System with Online Identification

**Figure 17:**
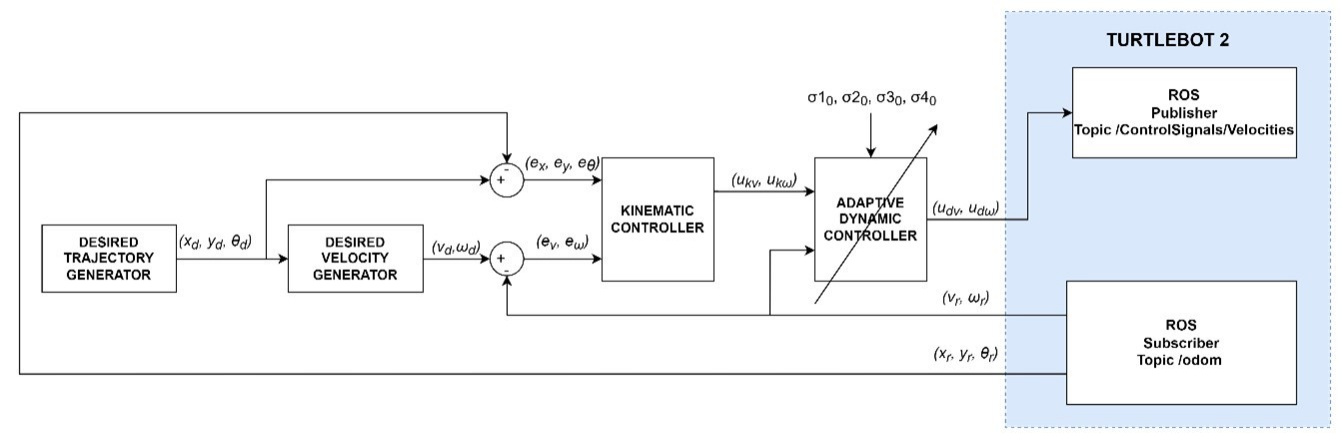
Block diagram of a real robot tracking motion with an adaptive controller.

The final step was to perform laboratory tests to identify the parameters of the robot’s dynamics model using online methods. A block diagram of trajectory tracking by the real TURTLEBOT 2 robot including the adaptive controller is shown in Figure 17.

**Figure 18:**
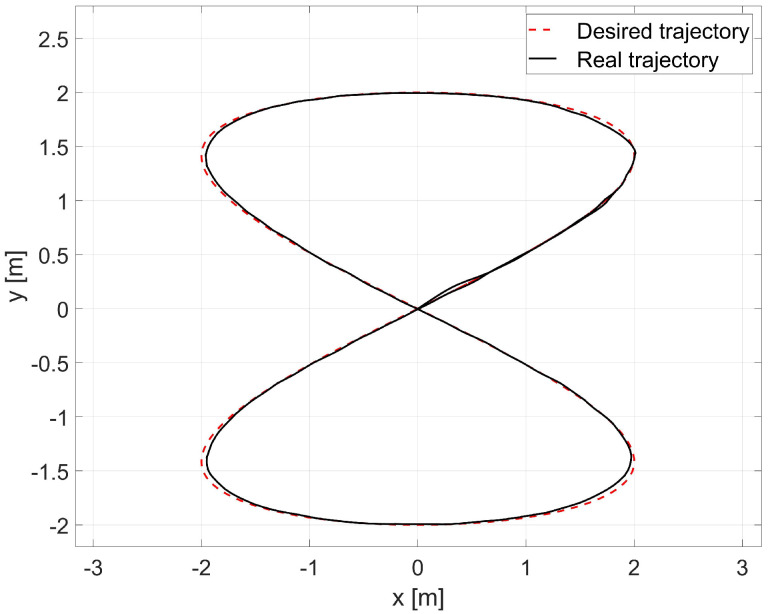
Comparison of reference and real trajectories in a laboratory test of RLS identification with random values of initial parameters.

**Figure 19:**
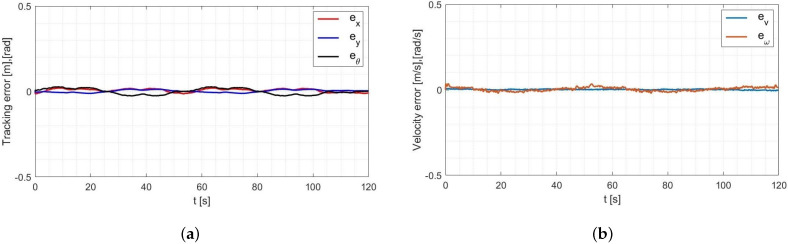
The errors of: (**a**) tracking the reference trajectory (**b**) speed.

**Figure 20:**
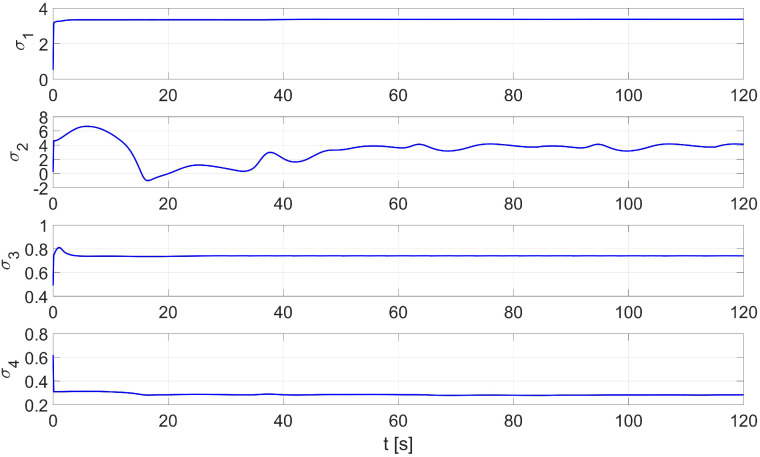
Distribution of parameters identified online by the RLS method with random values of initial parameters in the laboratory test.

The simulation was first carried out for random starting parameters with values of $\sigma_{1}=0.52,\;\sigma_{2}=0.23,\;\sigma_{3}=0.49,\;\sigma_{4}=0.62$. A comparison of the desired and resulting trajectories is shown in Figure 18. Position and orientation errors are shown in Figure 19a, while speed errors are shown in Figure 19b. The distribution of parameters identified during movement is shown in Figure 20.

Based on the analysis of the errors determined during the laboratory test for the RLS method with random initial parameters (Figure 19), it can be concluded that the adaptive controller, realizes control in tracking motion with position, orientation and velocity errors no greater than:

- in the x-axis direction: $e_{x}=0.021\;\left[m\right]$,
- in the y-axis direction: $e_{y}=0.013\;\left[m\right]$,
- orientation: $e_{\theta }=0.025\;\left[\mathrm{rad}\right]$,
- linear velocity $e_{v}=0.011\;\left[\frac{m}{s}\right]$,
- angular velocity $e_{\omega }=0.033\;\left[\frac{\mathrm{rad}}{s}\right]$.

The regulation time for the case of an 8-shape trajectory tracking task is equal to $t_{r}\;\approx \;7.5\;\left[s\right]$.

**Figure 21:**
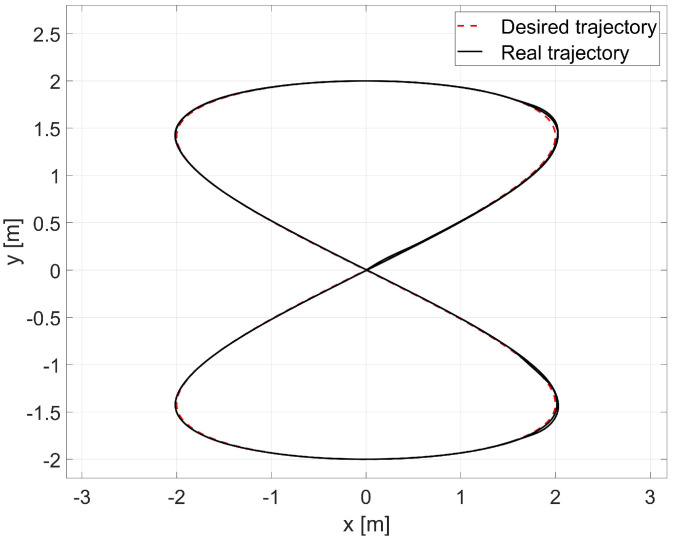
Comparison of the reference and real trajectories in the laboratory test of identification by the RLS method with the values of the initial parameters determined by the Levenberg-Marguardt method.

**Figure 22:**
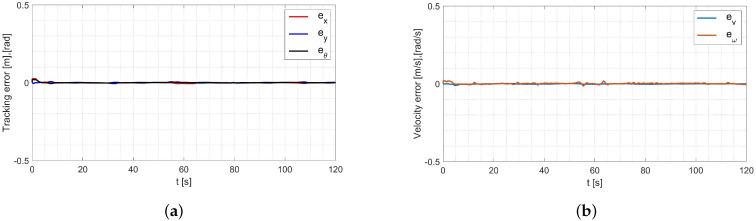
Errors for the RLS method with initial parameters determined by the Levenberg-Marguardt method: (**a**) tracking of the reference trajectory, (**b**) velocity.

**Figure 23:**
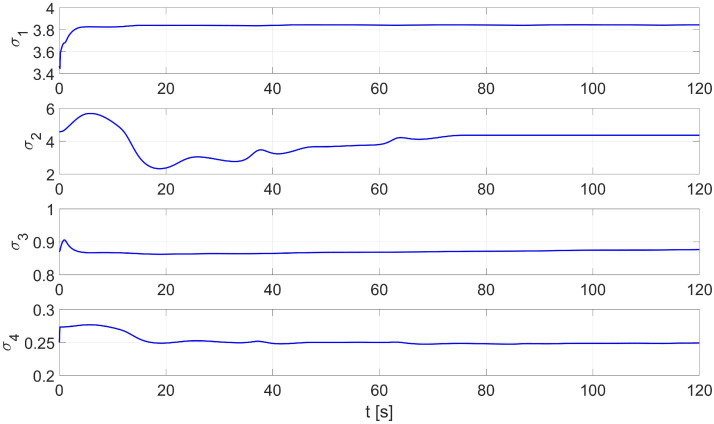
Distribution of parameters identified online by the RLS method with random values of initial parameters in the laboratory test.

The random initial parameters were then replaced with average values determined using the Levenberg-Marguardt method. A comparison of the desired and real trajectory is shown in Figure 21. The position and orientation errors for the combination of the RLS and Levenberg-Marguardt methods are shown in Figure 22a, while the velocity errors are shown in Figure 22b. The distribution of parameters identified during movement is shown in Figure 23.

Based on the analysis of the errors determined during the laboratory test for the RLS method with the initial parameters determined by the Levenberg-Marguardt method (Figure 22), it can be concluded that the adaptive controller, realizes control in tracking motion with position, orientation and velocity errors no greater than:

- in the x-axis direction: $e_{x}=0.009\;\left[m\right]$,
- in the y-axis direction: $e_{y}=0.008\;\left[m\right]$,
- orientation: $e_{\theta }=0.012\;\left[\mathrm{rad}\right]$,
- linear velocity $e_{v}=0.001\;\left[\frac{m}{s}\right]$,
- angular velocity $e_{\omega }=0.033\;\left[\frac{\mathrm{rad}}{s}\right]$.

The regulation time for the case of an 8-shape trajectory tracking task is equal to $t_{r}\;\approx \;1.6\;\left[s\right]$.

To compare the selected identification methods, all determined errors discussed in this article, are summarized in the Table 4 below:

## 5. Conclusions

The article presents the process of identifying the dynamics model parameters of a mobile robot on the example of the laboratory robot TURTLEBOT 2. The identification was carried out by an offline method using a preregistered trajectory and an online method—identifying the parameters during movement. The correctness of the identification process and the values of the identified parameters were checked first by simulating the robot’s tracking motion along a desired trajectory, and then by conducting laboratory tests using a real robot. By making a comparative analysis of the desired and simulation-determined trajectories, and during laboratory testing, it has been concluded that:

- all the values of the parameters identified by the selected methods are correct, and their implementation in the control system gives satisfactory results of tracking the desired trajectory;
- parameters identified by the Levenberg-Marguardt method allow trajectory tracking with a tracking error of about 30–40 [mm] and a regulation time of about 2.9 [s];
- the use of online identification by the RLS method with random initial parameters made it possible to achieve a tracking accuracy of the set trajectory of about 10–20 [mm] and a control time of 7.5 [s]; the use of adaptive technology in the control system made it possible to improve the tracking accuracy compared to the control system with constant values of the parameters of the robot model;
- The best tracking accuracy of the desired trajectory (a tracking error of about 2–5 [mm] and a regulation time of 1.6 [s]) was achieved using the RLS method with the initial parameters determined by the Levenberg-Marguardt method.

The conducted research has shown an improvement in the accuracy of trajectory tracking of AGV robots, by using a novel combination of offline and online methods proposed by authors, for the identification of mathematical model parameters. Since the identification process and control system have been developed for a differentially driven two-wheeled robot, the presented solution can be implemented to other AGV robots, with a drive system based on the one presented in the article. Since the TURTLEBOT 2 robot is not an accurate industrial unit (it is only a low-cost laboratory robot), it introduces errors in the form of vibrations and inaccuracies in measuring the rotation angle in the z-axis and y-axis (Figure 2a). Implementation of the methodology presented in this paper on a professional and more rigid robot could achieve even better accuracy in tracking the desired trajectory. The development trend toward the use of AGV robots in internal transportation and the cooperation of industrial and mobile robots in manufacturing processes in the age of Industry 4.0 confirms the advisability of the presented research and the need to develop methods to improve the accuracy of tracking the desired trajectory. 

## Figures and Tables

**Table 1:** Example values of parameters identified by the Levenberg-Marguardt method.

Test No.		$\sigma_{1}$	$\sigma_{2}$	$\sigma_{3}$	$\sigma_{4}$	Identification Time [s]
1	Initial values	0.16	0.52	0.96	0.37	
	Identified values	4.09	5.46	0.97	0.29	6.65
2	Initial values	0.61	0.26	0.76	0.29	
	Identified values	3.14	4.06	0.81	0.24	6.01
3	Initial values	0.59	0.55	0.92	0.29	
	Identified values	3.14	4.17	0.83	0.24	6.40
4	Initial values	0.65	0.45	0.55	0.30	
	Identified values	3.49	4.63	0.85	0.24	5.92
5	Initial values	0.38	0.56	0.08	0.05	
	Identified values	3.37	4.56	0.84	0.26	5.65
	Average	3.47	4.58	0.87	0.25	6.13
	Variance	0.15	0.30	0.00	0.00	
	Standard deviation	0.35	0.49	0.06	0.02	

**Table 2:** Example values of model parameters identified by the RLS method with random values of initial parameters.

Test No.		$\sigma_{1}$	$\sigma_{2}$	$\sigma_{3}$	$\sigma_{4}$
1	Initial values	0.6	0.71	0.22	0.12
	Identified values	3.16	4.15	0.92	0.3
2	Initial values	0.93	0.73	0.49	0.58
	Identified values	3.26	4.51	0.89	0.22
3	Initial values	0.24	0.46	0.96	0.55
	Identified values	3.84	4.34	0.86	0.25
4	Initial values	0.52	0.23	0.49	0.62
	Identified values	3.37	3.87	0.78	0.28
5	Initial values	0.68	0.39	0.37	0.99
	Identified values	3.32	4.16	0.76	0.24
	Average	3.39	4.21	0.84	0.26
	Variance	0.06	0.05	0.00	0.00
	Standard deviation	0.24	0.21	0.06	0.03

**Table 3:** Values of parameters identified by offline-assisted RLS methods.

		$\sigma_{1}$	$\sigma_{2}$	$\sigma_{3}$	$\sigma_{4}$
**Test No.**	Initial values	3.47	4.58	0.87	0.25
1	RLS + offline	3.68	4.49	0.90	0.22
2	RLS + offline	3.66	4.50	0.80	0.22
3	RLS + offline	3.62	4.51	0.85	0.23
4	RLS + offline	3.67	4.52	0.89	0.20
5	RLS + offline	3.62	4.54	0.83	0.24
	Average	3.65	4.51	0.85	0.22
	Variance	0.00	0.00	0.00	0.00
	Standard deviation	0.03	0.02	0.04	0.01

**Table 4:** A summary of the errors determined in the studies presented.

Errors	Simulation Tests			Laboratory Tests		
	L-M	RLS	RLS + L-M	L-M	RLS	RLS − L-M
$e_{x}\;\left[m\right]$	0.007	0.009	0.005	0.033	0.021	0.009
$e_{y}\;\left[m\right]$	0.007	0.016	0.002	0.046	0.013	0.008
$e_{\theta }\;\left[\mathrm{rad}\right]$	0.021	0.022	0.001	0.2	0.025	0.012
$e_{v}\;\left[\frac{m}{s}\right]$	0.001	0.001	0.001	0.023	0.011	0.001
$e_{\omega }\;\left[\frac{\mathrm{rad}}{s}\right]$	0.007	0.015	0.017	0.18	0.033	0.033
$t_{r}\;\left[s\right]$	8.2	7.8	4.8	2.9	7.5	1.6

## Abbreviations

The following abbreviations are used in this manuscript:

RLS	Recursive Least Squares method
L-M	Levenberg-Marguardt method
$x_{d},y_{d},\theta_{d}$	desired robot position and orientation
x,y,θ	desired robot position and orientation
$v_{d},\omega_{d}$	desired robot velocities (linear and angular)
v,ω	real robot velocities (linear and angular)
$v_{x},v_{y}$	forward and lateral robot velocities
$F_{r}rx,F_{r}ry$	longitudal and lateral force of right wheel
$F_{r}lx,F_{r}ly$	longitudal and lateral force of left wheel
*m*	mass of the robot
$I_{z}$	moment of inertia of the robot with respect to the axis passing through the point G
*G*	robot center of mass and center of rotation
$\sigma_{1},\ldots ,\sigma_{4}$	parameters of the robot dynamic model
$e_{x},e_{y},e_{\theta }$	position and orientation tracking errors
$e_{v},e_{\omega }$	velocity errors
$u_{k}v,u_{k}\omega$	control signals from kinematic controller
$k_{1},k_{2},k_{3}$	gains of the kinematic kontrollers
ϵ	oscillation damping coefficient
$\omega_{n}$	characteristic frequency
*b*	additional control coefficient
$u_{d}v,u_{d}\omega$	control signals from dynamic controller
$k_{v},k_{\omega }$	gains of the dynamic controller
$t_{r}$	regulation time

## Appendix A

In the case shown, the robot’s rotation point *G* is located on the axis connecting the drive wheels so the forces acting in the y-axis direction do not cause any torque. Therefore, the system of Equation (2) can be transformed to the form:

(A1) $$\begin{array}{c} {\dot{v}}_{x}=\frac{F_{rlx}+F_{rrx}}{m}+v_{y}\omega \\ \dot{\omega }=\frac{d\left(F_{rrx}-F_{rlx}\right)}{2I_{z}} \end{array}$$

Longitudinal velocity $v_{x}$, lateral velocity $v_{y}$ and angular velocity ω without taking slip into account are equal:

(A2) $$\begin{array}{c} v_{x}=\frac{1}{2}r\left(\omega_{r}+\omega_{l}\right)\\ v_{x}=0\\ \omega =\;\frac{1}{2d}r\left(\omega_{r}-\omega_{l}\right) \end{array}$$

where *r*—radius of driving wheels, $\omega_{r}$, $\omega_{l}$—angular velocity of wheels (right and left).

The angular velocity of the wheels can be determined by transforming the expressions for the right motor torques $\tau_{r}$ and left motor torques $\tau_{l}$ [57]:

(A3) $$\begin{array}{c} \tau_{r}=\frac{k_{a}\left(V_{r}-k_{b}\omega_{r}\right)}{R_{a}}\\ \tau_{l}=\frac{k_{a}\left(V_{l}-k_{b}\omega_{l}\right)}{R_{a}} \end{array}$$

where $V_{r},V_{l}$—the supply voltages of the motors (right and left), $k_{b}$—the electrical constants of motors, $k_{a}$—the mechanical constant of the engines, $R_{a}$—resistance of motor. The driving wheel dynamics equations [60] take the form:

(A4) $$\begin{array}{c} I_{e}\dot{\omega_{r}}+B_{e}\omega_{r}=\tau_{r}-F_{rrx}r\\ I_{e}\dot{\omega_{l}}+B_{e}\omega_{l}=\tau_{l}-F_{rlx}r \end{array}$$

where $I_{e}$—moment of inertia of the wheel with respect to the axis of rotation, $B_{e}$—coefficient of viscous friction reduced to the motor shaft. The equations of the PD controller implemented to regulate the supply voltage of motors [60] take the form:

(A5) $$\begin{array}{c} u_{v}=k_{PT}\left(v_{d}-v_{x}\right)-k_{DT}{\dot{v}}_{x}\\ u_{\omega }=k_{PR}\left(\omega_{d}-\omega \right)-k_{DR}\dot{\omega } \end{array}$$

where $u_{v}=\frac{V_{r}+V_{l}}{2},u_{\omega }=\frac{V_{r}-V_{l}}{2},k_{PT},\;k_{DT},\;k_{PR},\;k_{DR}$—gains of PD controllers, $v_{d}$—desired linear velocity, $\omega_{d}$—desired angular velocity.

By substituting Equation (A3) into Equation (A4) and transforming to form for determining longitudinal driving forces, a system of equations was obtained:

(A6) $$\begin{array}{c} F_{rrx}=\frac{1}{r}\left(\frac{k_{a}}{R_{a}}\left(V_{r}-k_{b}\omega_{r}\right)-I_{e}{\dot{\omega }}_{r}+B_{e}\omega_{r}\right)\\ F_{rlx}=\frac{1}{r}\left(\frac{k_{a}}{R_{a}}\left(V_{l}-k_{b}\omega_{l}\right)-I_{e}{\dot{\omega }}_{l}+B_{e}\omega_{l}\right) \end{array}$$

Then substituting Equation (A6) into the system of Equation (A1) obtained:

(A7) $$\begin{array}{c} {\dot{v}}_{x}=\frac{1}{rm}\left(\frac{k_{a}}{R_{a}}\left(V_{l}+V_{r}\right)+\left(\omega_{l}+\omega_{r}\right)\left(-\frac{k_{a}}{R_{a}}+B_{e}\right)-I_{e}\left(\dot{\omega_{l}+\omega_{r}}\right)\right)+v_{y}\omega \\ \dot{\omega }=\frac{d}{{I_{z}}^{2}r}\left(\frac{k_{a}}{R_{a}}\left(V_{r}-V_{l}\right)+\left(\omega_{r}+\omega_{l}\right)\left(-\frac{k_{a}}{R_{a}}k_{b}+B_{e}\right)-I_{e}\left(\dot{\omega_{r}+\omega_{l}}\right)\right) \end{array}$$

Combining the above equations with Equations (A2) and (A5) and performing algebraic transformations, expressions describing the TURTLEBOT 2 robot dynamics model were obtained:

(A8) $$\begin{array}{c} {\dot{v}}_{x}\left(\frac{1}{k_{PT}}\left(\frac{R_{a}}{k_{a}}\left(\frac{rm}{2}+\frac{I_{e}}{r}\right)+k_{DT}\right)\right)=-v_{x}\left(1-\frac{1}{rk_{PT}}\left(1+B_{e}\frac{R_{a}}{k_{a}}\right)\right)+v_{d}\\ {\dot{\omega }}_{x}\left(\frac{1}{k_{PR}}\left(\frac{R_{a}}{2k_{a}}\left(\frac{{I_{z}}^{2}r}{d}+\frac{{dI}_{e}}{r}\right)+k_{DR}\right)\right)=-\omega \left(\frac{d}{{rk}_{PR}}\left(1-\frac{R_{a}}{k_{a}}\right)+1\right)+\omega_{d} \end{array}$$

or in shortened form:

(A9) $$\begin{array}{c} {\dot{v}}_{x}\sigma_{1}=-v_{x}\sigma_{3}+v_{d}\\ {\dot{\omega }}_{x}\sigma_{2}=-\omega \sigma_{4}+\omega_{d} \end{array}$$

## Appendix B

Multiplying Equation (3) on both sides by $\left[\begin{array}{cc} \sigma_{1} & 0\\ 0 & \sigma_{2} \end{array}\right]$ the form was obtained:

(A10) $$\left[\begin{array}{cc} \sigma_{1} & 0\\ 0 & \sigma_{2} \end{array}\right]\;\left[\begin{array}{c} {\dot{v}}_{x}\\ \dot{\omega } \end{array}\right]=\left[\begin{array}{c} -\sigma_{3}v_{x}\\ -\sigma_{4}\omega \end{array}\right]+\left[\begin{array}{cc} 1 & 0\\ 0 & 1 \end{array}\right]\;\left[\begin{array}{c} v_{d}\\ \omega_{d} \end{array}\right]$$

By performing transformations, the above expression can be written in the form:

(A11) $$\left[\begin{array}{cc} \sigma_{1} & 0\\ 0 & \sigma_{2} \end{array}\right]\;\left[\begin{array}{c} {\dot{v}}_{x}\\ \dot{\omega } \end{array}\right]+\left[\begin{array}{c} \sigma_{3}\\ \sigma_{4} \end{array}\right]\left[\begin{array}{cc} v_{x} & 0\\ 0 & \omega \end{array}\right]=\left[\begin{array}{cc} 1 & 0\\ 0 & 1 \end{array}\right]\;\left[\begin{array}{c} v_{d}\\ \omega_{d} \end{array}\right]$$

and then bring it to parametric form:

(A12) $$\left[\begin{array}{c} v_{d}\\ \omega_{d} \end{array}\right]=\left[\begin{array}{cc} \sigma_{1} & 0\\ 0 & \sigma_{2} \end{array}\right]\;\left[\begin{array}{c} {\dot{v}}_{x}\\ \dot{\omega } \end{array}\right]+\left[\begin{array}{cc} \begin{array}{cc} \begin{array}{c} 0\\ 0 \end{array} & \begin{array}{c} 0\\ 0 \end{array} \end{array} & \begin{array}{cc} \begin{array}{c} v_{x}\\ 0 \end{array} & \begin{array}{c} 0\\ \omega \end{array} \end{array} \end{array}\right]{\left[\begin{array}{cc} \begin{array}{ccc} \sigma_{1} & \sigma_{2} & \sigma_{3} \end{array} & \sigma_{4} \end{array}\right]}^{T}$$

or in shortened form:

(A13) $$\left[\begin{array}{c} v_{d}\\ \omega_{d} \end{array}\right]=\left[\begin{array}{cc} \begin{array}{cc} \begin{array}{c} {\dot{v}}_{x}\\ 0 \end{array} & \begin{array}{c} 0\\ \dot{\omega } \end{array} \end{array} & \begin{array}{cc} \begin{array}{c} v_{x}\\ 0 \end{array} & \begin{array}{c} 0\\ \omega \end{array} \end{array} \end{array}\right]{\left[\begin{array}{cc} \begin{array}{ccc} \sigma_{1} & \sigma_{2} & \sigma_{3} \end{array} & \sigma_{4} \end{array}\right]}^{T}$$

(A14) $$\left[\begin{array}{c} v_{d}\\ \omega_{d} \end{array}\right]=A\sigma$$

Based on the inverse dynamic task [61], the above expression (A12) can be reduced to the form of dynamic controller (12).
